# Uncovering associations between interest in One Health and pre-existing conditions and behaviours: Evidence from a UK survey

**DOI:** 10.1016/j.onehlt.2024.100732

**Published:** 2024-04-16

**Authors:** Elin Pöllänen, Timothy Yu-Cheong Yeung, Jane Arroyo, Hyo Won Park, Carolin Formella, Walter Osika

**Affiliations:** a Karolinska Institutet, Stockholm, Sweden; b Centre for European Policy Studies, Brussels, Belgium

**Keywords:** One health, Mental health, Human-animal relations, Nature connection, Health policies, Health governance

## Abstract

This paper endeavours to unveil individual characteristics associated with an interest in One Health. Through the distribution of an online survey randomly distributed among the United Kingdom population, we discovered significant correlations between pre-existing attitudes towards and relationships with nature and animals and interest in One Health, which is quantified by the number of additional pages of One Health information participants agreed to view at the survey's conclusion. Additionally, individuals with poorer mental health demonstrated a higher level of interest in One Health. The findings suggest that interest in One Health and people's connections with nature and animals are driven by the same personal preferences. These insights point towards the potential for more targeted communication strategies to specific groups, facilitating more effective promotion of the One Health concept.

## 1 Introduction

Most human infectious diseases are considered to have a zoonotic origin, such as the severe acute respiratory syndrome (SARS), H1N1 influenza, Middle East Respiratory Syndrome (MERS), Zika and Ebola [1,2]. Most recently, the Covid-19 pandemic has showcased the devastating impact an infectious disease outbreak can have on various facets and levels of society, and the vulnerabilities that exist.

Land-use change and deforestation, wildlife trade, and intensified livestock production are all examples of zoonotic drivers, activities that increase the risk of disease transmission from animals to humans [3,4] By increasing the interaction between humans and different species, it is suggested that these activities will make spillover events more frequent [2]. Public health crises driven by outbreaks of infectious diseases have caused stakeholders to recognise the need for a greater interdisciplinary collaboration to prevent and control zoonoses. The One Health framework is an approach that aims to sustainably balance and optimise the health of humans, animals, and ecosystems, acknowledging that they are interconnected and interdependent [5,6]. Following the Covid-19 pandemic, the One Health approach has gained momentum in global health governance. For example, to improve preparedness and response to health crises, the European Union adopted a health legislative package in 2022 for a “European Health Union” [7], which includes adopting a “One Health approach” in its health preparedness and response as well as in its training activities as stated in Regulation (EU) 2022/2371.

The main challenges with One Health include its vague conceptualization, a lack of translation from theory to practice, a narrow and reactive stance that acts only after pathogens have emerged and pose a threat to humans, and existing barriers and imbalances between the three sectors [[8], [9], [10], [11], [12]]. To make One Health a more tangible concept, the One Health High-Level Expert Panel (OHHLEP), an advisory group for Quadripartite organisations^1^ recently created a working definition of One Health as well as guiding principles to help with implementation [5]. Whilst there has been an interest in researching the perception and framing of One Health amongst practitioners and collaborators [13], the awareness of One Health amongst the public is considered low [12] and peoples' willingness to engage in and support One Health related activities, remain to be further explored.

Previous studies have found human relationships to (other) animals to be largely shaped by (sociocultural) human identity and particularly attitudes such as human exceptionalist and prejudice towards animals [14,15] and impacted by several factors such as gender and diet [16,17]. For instance, a plant-based diet has shown to indicate a higher animal welfare attitude and compassion towards animals [18]. Furthermore, earlier research has indicated that more contact with green areas and a sense of connection to nature are amongst the factors associated with stronger pro-environmental attitudes and actions [[19], [20], [21]]. Framing animals and natural environments as carriers of pathogens and possible threats to human health, as One Health often does, fails to acknowledge mutual health benefits and can therefore negatively impact public attitudes for policies aimed more at improving animal and environmental health [13,22]. A study on One Health messaging about bats and rabies suggest that if a risk perspective is used (for example), it should be complemented with information on the benefits of bats or anthropocentric factors that drive health risks to increase support of One Health and wildlife conservation goals [23]. According to another study on the impact of framing on public support for environmental management, intrinsic environmental worth and preventing further environmental degradation were shown to be more motivating than economic benefit and additional environmental gains [24].

A deeper understanding of people's interest in One Health can contribute to the research field of One Health implementation as well as public health and sustainability initiatives overall, and identify the circumstances when people feel interconnected to, and are willing to act in solidarity with, more-than-human beings. For instance, it has been proposed that more-than-human solidarity (acknowledgment of commitment to places and other species) could help develop the public health ethics of One Health [25] and help create legitimisation for much needed efforts that tackle underlying drivers of disease [4,26]. In its current form, in contrast to a “bolder”, “wider”, or more “radical” version [9,[27], [28]], One Health is narrow and human-centric, and more might be required to make people develop more-than-human solidarity.

As many diseases have global implications but begin locally, it has been suggested that a top-down approach should be complemented with an increased understanding and inclusion of local communities' knowledge, attitudes, and practices related to One Health topics, which could empower and engage local communities to act as agents of change and prevent outbreaks [[29], [30]]. Yet a unilateral focus on a bottom-up approach could also lead to biased decision-making. Authorities need a balanced sampling as well as understanding of real-world evidence to better integrate the One Health framework into public health policies.

A better understanding of people's preferences, and their associations with people's characteristics, will help policymakers and One Health scholars to design targeted communication and the context for general acceptance for wider One Health policies. This study therefore aimed to investigate the interest in One Health amongst individuals, and what factors might impact this interest or non-interest.

## 2 Material and methods

To identify associations of interest in One Health and individual characteristics, a survey was designed aiming to discover any correlations between respondents' characteristics and their attitudes towards animals, the environment, and the One Health concept. A marketing company, Panelbase, was hired to distribute the survey to its platform users in the United Kingdom between June 2023 and August 2023. The survey was designed in English, absorbing insights from the relevant literature of the same language to ensure comparable data and analysis across research. The UK was chosen as the only country for the study, a decision based on the language of the survey and comparability, as well as on the assumption that a random sample of the population would interpret the context and terminology with similar linguistic understanding. The distribution was random but aimed to include an adequate number of subjects in each age group. In total, 1287 effective responses were received, in which 95 respondents claimed to have heard of One Health. As no other conditions were specified except for age, the sample is believed to be a randomly selected subset of a population. Yet, respondents might self-select into first the network of the marketing company and second responding to the survey.

### 2.1 Independent variables

Background information collected through this survey includes:

1. Gender
2. Highest level of education
3. Age group
4. Whether one owns/owned a pet
5. Diet type
6. Frequency of visiting green spaces
7. Urban/Rural residence

Also, the mental health conditions of respondents were collected by using the Patient Health Questionnaire-4 (PHQ-4), which measures respondents' anxiety and depression level [31].^2^ The index is built upon four 4-point Likert scale questions and can range from 0 to 12. The higher the score the more severe anxiety and depression one experienced.

### 2.2 Dependent variables

One Health was measured by a behavioural observation. At the end of the survey, respondents were asked if they would like to know more about One Health. The respondents were showed some brief information about One Health and then asked if they wanted to know more. If the respondent responded “not interested”, the survey would arrive at an end. If the respondent responded “interested”, the survey would show another page of information of One Health. In total there were three such questions and thus four pages of information about One Health. Therefrom, each respondent became paired with a score ranging from 0 to 3 that objectively measured their interest in One Health. Due to this construction of the measure of interest in One Health, those who claimed to have heard of One Health are excluded from the analysis.

**Fig. 1:**
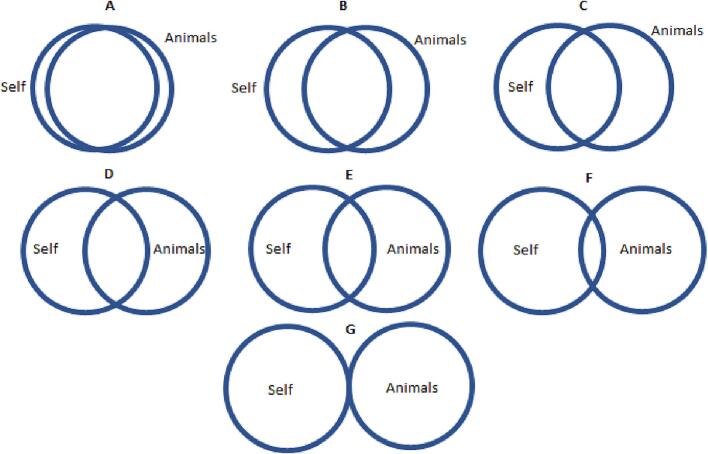
Venn diagram: which picture best describes your relationship with animals?

**Fig. 2:**
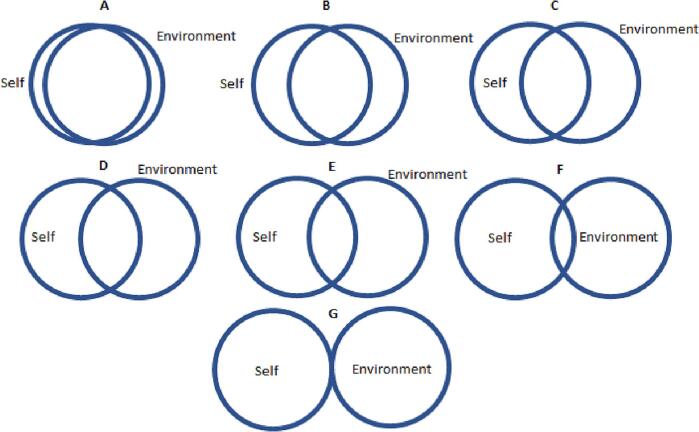
Venn diagram: which picture best describes your relationship with the environment?

Additional to the behavioural observation, respondents' self-reported attitudes towards animals and the environment were collected by using four measures. To measure respondents' attitude towards animals, the Human Supremacy Belief [14] was used, which contains six items on a 7-point Likert scale version, with score ranging from 0 to 36. Attitudes towards the environment was measured by using the Willingness to Sacrifice index [32]. The measure contains five items, all normally scored. To maintain consistency, the 7-point Likert scale version of the measure was used, which also ranges from 0 to 36. Also, two simple measures were developed and included: seven Venn diagrams each containing two circles where the overlapping between areas decreased from the first to last diagram, as shown in Fig. 1 and Fig. 2. This was meant to capture to which extent respondents felt “overlapped” with animals and the environment. These measures were all expected to be highly correlated with the respondent's interest in One Health and serve as alternative dependent variables for checking the robustness of the results.

To compute the correlations between characteristics and interest in One Health, a multi-variate regression analysis was applied. Note that the results should only be interpreted as correlations, and the underlying mechanisms and causal relationships can only be speculations.

Together with those who claimed to have heard of One Health, respondents giving uninformative answers are excluded. The subsequent analysis thus includes the information provided by 1181 subjects.

## 3 Results

Table 1 presents the correlation coefficients between the five dependent variables. The Interest in One Health and the two ‘overlap’ measures are moderately correlated with the two established measures, i.e. Human Supremacy Belief [14] and Willingness to Sacrifice for the environment [31].

**Table 1:** - Correlation matrix of dependent variables.

Variable	Interest OH	Human Supremacy	Willingness to sacrifice	Animal overlap	Environment overlap
Interest OH	1				
Human supremacy	−0.2697	1			
Willingness to sacrifice	0.3820	−0.4758	1		
Animal overlap	0.2488	−0.5323	0.3611	1	
Environment overlap	0.2858	−0.3613	0.6026	0.5002	1

Table 2 presents the multi-variate regression analysis, which applies the Ordinary Least Square model. Column 1 presents the regression result with interest in One Health as the dependent variable. Notably, the poorer the mental health the higher the interest in One Health ($\hat{\beta }=0.034$; *p*-value = 0.011). Owning a pet is associated with higher interest in One Health, compared to those who have never owned a pet ($\hat{\beta }=-0.300$; p-value = 0.027). Being partial vegetarian ($\hat{\beta }=0.525$; p-value = 0.000) and lifestyle vegan ($\hat{\beta }=0.605$; p-value = 0.013) are associated with higher interest in One Health than being omnivorous. More frequent visitors of green space are also associated with higher interest in One Health. Meanwhile, no significant correlations can be found between interest in One Health and age, gender, educational attainment or residence. Column 2 to 5 report the results of the same model but replacing the interest in One Health by four other measures of attitudes. Column 2 shows that male subjects tend to associate with a higher human supremacy score, which is consistent with the result shown in Column 3 where animal overlap score is the dependent variable. Interestingly, frequent green space visitors do not show significant lower human supremacy tendency.

**Table 2:** - Multi-variate regression analysis.

		(1)	(2)	(3)	(4)	(5)
Dep. Variable	No. of obs.	Interest in One Health	Human Supremacy	Animal overlap	Willingness to sacrifice	Environment overlap
PHQ-4		0.034** (0.013)	−0.191*** (0.068)	0.052*** (0.016)	−0.016 (0.058)	−0.019 (0.016)
Gender (Ref: Female)						
Male	549	−0.034 (0.085)	2.225*** (0.414)	−0.319*** (0.097)	−1.388*** (0.374)	−0.181* (0.094)
Age (Ref: 18-30)						
31–40	215	−0.068 (0.151)	1.216 (0.752)	0.170 (0.163)	0.069 (0.627)	0.367** (0.155)
41–50	227	0.113 (0.148)	0.242 (0.762)	0.253 (0.161)	−0.049 (0.622)	0.198 (0.159)
51–60	198	0.204 (0.154)	0.234 (0.780)	0.248 (0.172)	−0.047 (0.662)	0.317* (0.170)
61–70	232	0.046 (0.156)	0.088 (0.780)	0.397** (0.173)	0.616 (0.622)	0.474*** (0.167)
71+	158	−0.010 (0.172)	−0.163 (0.844)	0.425** (0.195)	0.954 (0.717)	0.380** (0.184)
Education (Ref: Primary)						
Secondary	503	−0.199 (0.533)	−3.557 (2.373)	0.949 (0.638)	3.461 (3.413)	−0.102 (0.619)
Tertiary	461	−0.122 (0.533)	−3.698 (2.378)	0.784 (0.639)	4.701 (3.436)	0.200 (0.627)
Postgraduate	184	−0.043 (0.540)	−3.461 (2.425)	0.702 (0.647)	4.389 (3.418)	0.009 (0.620)
Others	26	0.152 (0.596)	−3.698 (2.777)	0.640 (0.714)	4.413 (3.704)	−0.058 (0.688)
Pet ownership (Ref: Currently own a pet)						
Owned one in the past	398	−0.087 (0.092)	1.434*** (0.462)	−0.980*** (0.106)	−0.658 (0.419)	−0.194* (0.103)
Never owned a pet	140	−0.300** (0.135)	2.749*** (0.656)	−1.736*** (0.149)	−1.333** (0.598)	−0.210 (0.148)
Diet (Ref: Omnivorous)						
Partial vegetarian	142	0.525*** (0.116)	−2.740*** (0.635)	0.349** (0.156)	2.852*** (0.496)	0.556*** (0.146)
Vegetarian	65	0.112 (0.181)	−5.704*** (0.979)	0.638*** (0.198)	3.877*** (0.743)	0.555*** (0.183)
Dietary vegan	7	0.352 (0.518)	−10.532*** (2.101)	1.321** (0.574)	3.014 (2.113)	0.670 (0.667)
Lifestyle vegan	23	0.605** (0.243)	−9.281*** (1.502)	1.755*** (0.262)	6.631*** (1.662)	1.390*** (0.335)
Prefer not to say	51	−0.340 (0.207)	−0.007 (1.010)	0.087 (0.245)	−0.530 (1.068)	0.045 (0.248)
Green space visits (Ref: Everyday)						
A few times a week	399	−0.063 (0.117)	0.246 (0.589)	−0.120 (0.134)	−0.302 (0.528)	−0.059 (0.130)
About once a week	205	−0.114 (0.138)	−0.143 (0.684)	−0.136 (0.157)	−0.529 (0.595)	−0.102 (0.151)
A few times a month	151	−0.323** (0.153)	1.258* (0.756)	−0.366** (0.172)	−1.599** (0.667)	−0.362** (0.161)
Once a month	46	−0.444* (0.233)	0.553 (1.234)	−0.333 (0.287)	−2.900** (1.208)	−0.686** (0.281)
Less than once a month	150	−0.432*** (0.156)	−0.380 (0.828)	−0.267 (0.193)	−1.730** (0.746)	−0.405** (0.181)
Residence (Ref: Urban)						
Suburban	619	0.050 (0.099)	−0.369 (0.496)	0.350*** (0.114)	−0.348 (0.444)	0.111 (0.111)
Countryside	239	0.155 (0.125)	−1.164* (0.654)	0.453*** (0.144)	−0.020 (0.568)	0.262* (0.140)
Others	11	0.047 (0.382)	3.242 (2.234)	−0.363 (0.539)	0.865 (1.433)	0.086 (0.391)
Constant		1.756*** (0.565)	23.907*** (2.562)	3.454*** (0.672)	21.646*** (3.502)	4.199*** (0.648)
R-squared		0.059	0.162	0.215	0.120	0.082
Total no. of Obs.		1181	1181	1181	1181	1181

Note: Robust standard errors are shown in parentheses. *Significant at 10%; **significant at 5%; ***significant at 1%.

Column 4 and Column 5 turn to attitudes towards the environment, where mental health is no longer a significant variable. Non-omnivorous dietary preferences and frequent green space visitors are associated with higher willingness to sacrifice for the environment and higher environment overlap scores.

When comparing the results of the five regressions, it can be interpreted that subjects differentiate animals from the environment, and vice versa, and that there are different mechanisms in place that influence human attitudes towards animals and towards the environment.

## 4 Discussion

This study attempts to identify associations between interest in One Health and individual background and behavioural traits through a UK-based on-line survey, and finds mental health, owing a pet, diet and frequency of green space visits associated with higher levels of interest in One Health.

Respondents identifying with having a vegan or partial vegetarian lifestyle show significantly higher interest in One Health compared to omnivores. Also, participants with current pet ownership have higher levels of interest in One Health compared with them who never had owned a pet. It is likely that an interest in One Health and some behavioural traits are driven by the same subjects' preferences. Previous research has shown that pet owners are more involved in animal protection and more in favour of awarding fundamental rights to animals [33]. Differences in people's values and empathy levels have been found regarding diet. Vegetarianism was linked to greater openness and empathy, and values such as universalism, hedonism, and self-direction. In contrast, omnivores to a higher extent manifested ideas of power, social dominance, prejudice and right-wing authoritarianism [34]. It is likely that the notions of connection and traits of openness and empathy increase a person's willingness to learn more and embrace a concept that is based on interconnection and cooperation across borders and disciplines.

The survey result indicates a relation between lower levels of mental well-being and higher levels of interest in One Health. Newer research has started to unravel the links between mental health and the ongoing and escalating climate crisis [35]. Higher levels of compassion towards others, and more sustainable attitudes and behaviours, have been related to lower levels of wellbeing [36], and engagement in climate issues has been shown to be related to grief and higher levels of anxiety and depression [[37], [38]]. Terminologies such as “eco-anxiety” and “climate anxiety” have been coined. In contrast to paralysing anxiety, triggering anxiety can create responses such as information gathering and behavioural change [39]. In burnout research, symptoms can be signals of a “sick system” and dysfunctional coping strategies, and the need for other, more benefitting structures [40]. Similarly, negative responses to the ongoing destruction of nature and mass suffering of animals could indicate lower cognitive dissonance (value-action gap) and a higher connection to the surrounding world, serving as mental manifestations of the human-animal-environmental bond and desire for structural changes. This result also points to the necessity of managing the mental health condition of the public together with enhancing awareness of the health of nature and animals.

This study has a few limitations. First, the sample is not a strictly random sample drawn from the population. The respondents were associated with the marketing company's network and their characteristics might not be comprehensively representative for the whole population. Yet given the distribution of the respondents across age ranges and education backgrounds, the quantitative analysis can be considered relevant for the UK population and other populations. Second, the study unavoidably relies on self-reported information, yet with efforts to amend the problem by employing a directly observed behavioural measure of interest in One Health. Third, as the study collected information from the UK population only, the generalizability to other populations is unknown. However, as discussed above, the insights offered by this study can still be relevant to other populations and countries as well. Fourth, the survey did not collect certain socio-economic factors, such as income level, which might be of relevance. Even so, the results point to some highly relevant research and policy questions, such as exploring ways in which One Health could aim to reach the public and how the messaging should be framed to reach different individuals and populations, without the risk of evoking or deepening existing stereotypical beliefs of human exceptionalism that increase the human-animal-environmental gap.

## 5 Conclusions

Utilising survey data and employing multivariate regression analysis, this study sheds light on critical implications for practical application. Notably, the findings underscore significant associations between individuals' existing relationships with nature and animals and their interest in One Health. Moreover, a positive association is observed between heightened interest in One Health and poorer mental health outcomes. This research highlights a significant knowledge gap pertaining to public awareness of the One Health concept and its potential advantages, alongside individual experiences with their environment and mental well-being.

Despite aspirations to integrate One Health principles into public health policies, challenges persist in translating theory into actionable measures. The study underscores the pressing need for empirical evidence and deeper understanding of public preferences and experiences to inform the development of One Health-centered policies. Future research endeavours should explore the capacity of the One Health framework to engage diverse stakeholders and foster understanding and acceptance of policies aimed at enhancing animal and environmental welfare. The Covid-19 pandemic starkly underscored the repercussions of overlooking the interconnectedness of human, animal, and environmental health, emphasizing the urgency for transformative action at all levels. Consequently, further research is imperative to comprehensively assess the potential of One Health to drive substantial reform rather than mere rhetorical gestures.

## CRediT authorship contribution statement

**Elin Pöllänen:** Conceptualization, Methodology, Writing – original draft, Writing – review & editing. **Timothy Yu-Cheong Yeung:** Conceptualization, Data curation, Methodology, Visualization, Writing – original draft, Writing – review & editing. **Jane Arroyo:** Data curation, Methodology. **Hyo Won Park:** Writing – original draft. **Carolin Formella:** Conceptualization, Data curation. **Walter Osika:** Conceptualization, Methodology, Writing – original draft, Writing – review & editing.

## Declaration of competing interest

None.

## Data Availability

Data will be made available on request.

## Footnotes

1 Consists of the Food and Agriculture Organization of the United Nations (FAO), United Nations Environment Programme (UNEP), World Health Organization (WHO) and World Organization for Animal Health (WOAH).

2 The PHQ-4 is available from www.phqscreeners.com free of charge, and permission for use is not required. See https://www.oregonpainguidance.org/app/content/uploads/2016/05/PHQ-4.pdf
